# Liquid Biopsy of Hepatocellular Carcinoma: Circulating Tumor-Derived Biomarkers

**DOI:** 10.1155/2016/1427849

**Published:** 2016-06-15

**Authors:** Chang-Qing Yin, Chun-Hui Yuan, Zhen Qu, Qing Guan, Hao Chen, Fu-Bing Wang

**Affiliations:** ^1^Department of Laboratory Medicine and Center for Gene Diagnosis, Zhongnan Hospital of Wuhan University, No. 169 Donghu Road, Wuchang District, Wuhan 430071, China; ^2^Department of Immunology, School of Basic Medical Sciences, Wuhan University, No. 185 Donghu Road, Wuchang District, Wuhan 430071, China

## Abstract

Hepatocellular carcinoma (HCC) is the second leading cause of cancer-related death worldwide due to latent liver disease, late diagnosis, and nonresponse to systemic treatments. Till now, surgical and/or biopsy specimens are still generally used as a gold standard by the clinicians for clinical decision-making. However, apart from their invasive characteristics, tumor biopsy only mirrors a single spot of the tumor, failing to reflect current cancer dynamics and progression. Therefore, it is imperative to develop new diagnostic strategies with significant effectiveness and reliability to monitor high-risk populations and detect HCC at an early stage. In the past decade, the potent utilities of “liquid biopsy” have attracted intense concern and were developed to evaluate cancer progression in several clinical trials. “Liquid biopsies” represent a series of noninvasive tests that detect cancer byproducts easily accessible in peripheral blood, mainly including circulating tumor cells (CTCs) and cell-free nucleic acids (cfNAs) that are shed into the blood from the tumor sites. In this review, we focus on the recent developments in the field of “liquid biopsy” as well as the diagnostic and prognostic significance of CTCs and cfNAs in HCC patients.

## 1. Introduction

Hepatocellular carcinoma (HCC) is the sixth most common cancer worldwide (782,451 cases in 2012, 5.6% of all cancers); however, it ranks as the second cause of cancer-related death (nearly 745,553 deaths in 2012, 9.1% of all cancers) [1, 2]. Owing to the characters of hepatocellular carcinoma, like insidious onset, high degree of malignancy, and nonspecific symptoms in early stage, the prognosis of HCC remains dismal with a 5-year overall survival rate of 0%–10% [3–5]. Conventional diagnostic methods for the detection of HCC include serological tests and imaging examination, however, which show less sensitivity and specificity, and most patients diagnosed with HCC are already in advanced stage or with underlying liver diseases [6]. Traditional computed tomography (CT) scan, for example, can be used to detect changes in tumor size but does not provide information regarding the biology of the tumor. Additionally, CT scan is typically examined in an interval of several months because it is not sensitive enough to detect minimal tumor volume changes in short period. Serological tests include *α*-fetoprotein (AFP), descarboxy prothrombin (DCP), and AFP-L3, which have been studied alone or in combination [7]. However, available data has shown that the elevation of such serum tumor markers is only weakly associated with the tumor progression and may be elevated for tumor-uncorrelated reasons. For example, AFP is the widely used molecular markers for clinical HCC diagnosis; however, it often showed a false-positive result during pregnancy, as well as active liver disease, embryonic tumor, and certain gastrointestinal tumors [7–9]. Therefore, to facilitate the development of “precision medicine” cancer management program, the importance of developing useful diagnostic and monitoring tools that provide timely and accurate information for an individual cancer patient should be emphasized.

**Figure 1:**
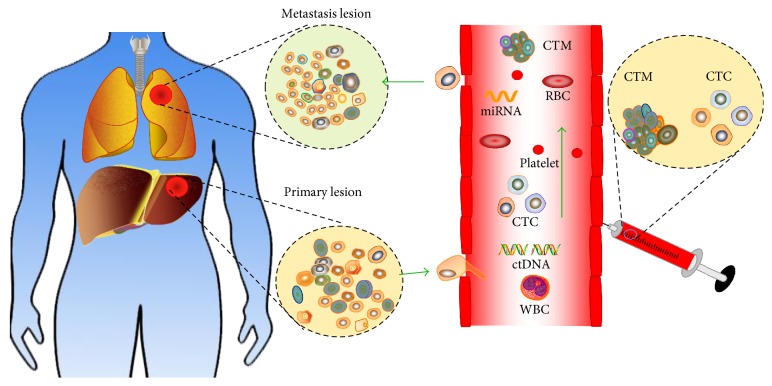
Liquid biopsy of hepatocellular carcinoma: circulating tumor cells (CTCs) and cell-free tumor-associated DNA or miRNAs (cfNAs) are easily accessible in peripheral blood of patients. Analysis of these cells or molecules can be used for early tumor detection and provide prognostic information for HCC patients.

In the past few decades, numerous studies have demonstrated the potent utility of circulating cancer byproducts detection (e.g., CTCs, cfNAs), namely, “liquid biopsy,” which could provide accessible, accurate, and dynamic information to evaluate tumor progression (Figure 1). Unlike a traditional biopsy, several advantages seem to contribute to the prevalence of “liquid biopsy”: (1) it is a noninvasive procedure that can be conducted routinely without any clinical difficulties; (2) it allows us to have repeated samplings which offer a multifaceted portrait of the disease over time, whereas tissue samples only give doctors a localized picture; (3) it is more likely to capture the overall genetic complexity of both primary and metastatic lesions in patients with advanced disease; and (4) it is much faster and cheaper than classical biopsy testing for early disease detection [10, 11].

Therefore, with the aim of gaining a better insight into the “liquid biopsy” in patients with HCC, we provide a comprehensive overview of the historical backgrounds, characterizations, and recent developments of both CTCs and cfNAs in patients with HCC and discuss future perspectives.

## 2. Circulating Tumor Cells (CTCs)

Circulating tumor cells are widely recognized as the “seeds” of tumors, which are shed into peripheral blood from carcinoma* in situ* and eventually establish metastatic carcinoma in other organs. The presence of CTCs was observed for the first time in 1869 in the blood of a man with metastatic cancer by Ashworth, who also hypothesized that they would throw some light on the origin of multiple tumors existing in the same person [12]. However, for over a century, CTC research has been hampered by the incapacity to detect these rare cells, which are estimated in frequencies of about 1–10 CTCs in a background of millions of blood cells in patients with metastatic disease [13]. Therefore, the major challenge for CTC researchers is the difficulty in CTC purification which directly determined the following molecular characterization and clinical evaluation of CTCs.

In recent years, many different CTC isolation technologies have emerged to improve CTC isolation or detection rates, and they are mainly categorized into two groups: physical methods and biological methods. The former methods mainly depend on the physical properties of CTCs, such as size, density, electric charge, migratory capacity, and deformability [44]. The biological methods mainly rely on antigen-antibody binding, and antibodies against tumor specific biomarkers including epithelial cell adhesion molecule (EpCAM), human epidermal growth factor receptor 2 (Her2), and prostate-specific antigen (PSA) are usually used in CTCs purification [45]. In particular, EpCAM is the most commonly used antigen in CTCs purification because its expression is virtually universal (albeit at variable levels) in cells of epithelial origin, but it is absent in blood cells [45, 46] and more importantly, up to now, EpCAM-based Cell-Search*™* (Veridex LLC, NJ, USA) is still the first and only clinically validated, FDA-approved test for capturing and enumerating CTCs to facilitate clinical decision-making. In this platform, tumor cells are first enriched immunomagnetically by EpCAM antibody-coupled magnetic beads. Subsequently, recovered cells are permeabilized and immunostained with a nuclear stain (4′,6′-diamidino-2-phenylindole hydrochloride; DAPI), a fluorescent antibody conjugate against CD45 (leukocyte marker), and cytokeratin 8, cytokeratin 18, and cytokeratin 19 (cytoplasmic epithelial markers) [47]. However, although immunocapture enrichment methods could acquire high purity of CTCs, such methods may miss the tumor cells with highly metastatic potential due to the epithelial-mesenchymal transition (EMT) process of CTCs, which is characterized by decreased expression of epithelial markers and the acquisition of mesenchymal features [48]. Therefore, alternative enrichment approaches should also take mesenchymal markers into account and avoid the biased capture of CTC population.

**Figure 2:**
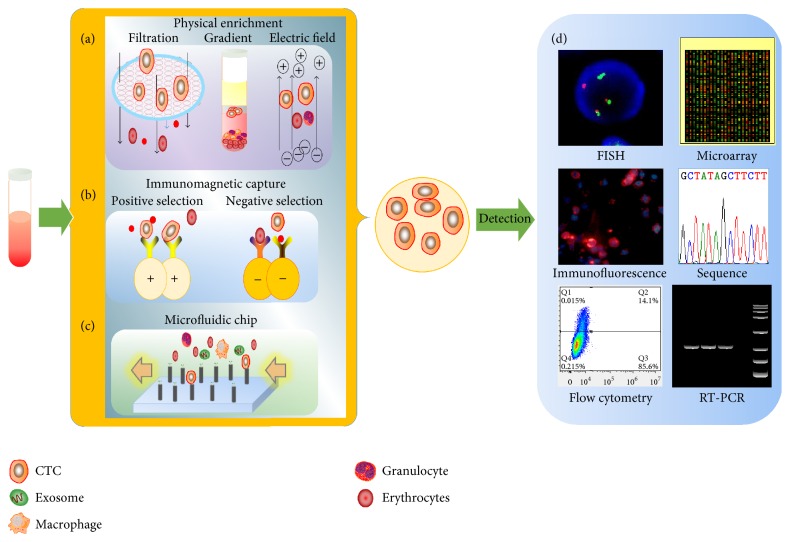
Schematic model of CTCs enrichment and further identification of molecular information. Enrichment of CTCs from the peripheral blood of cancer patients is based on the following principles: CTCs can be enriched on the basis of physical properties, including size (filtration-based devices), density (Ficoll centrifugation), and electric charges (dielectrophoresis) (a). CTCs can also be positively or negatively enriched on the basis of the expression of cell surface markers (EpCAM for positive selection and CD45 for negative selection) (b). CTCs can also be trapped from PBMCs by flowing through posts in a microfluidic chip on the basis of both physical and biological properties (c). The subsequent analysis of CTCs including fluorescence* in situ* hybridization (FISH), microarray, immunofluorescence, sequencing, flow cytometry, and RT-PCR (d).

Most recently, the microfluidic-based “CTC-chip” has attracted intense attention due to its enhanced sensitivity and specificity in CTCs purification as demonstrated by the accumulated evidence [49, 50]. In this platform, CTCs could be isolated from small volumes of blood samples by flow through “antibody”-coated microposts. And, compared with macroscale approaches, microfluidic devices offer multiple advantages for CTC isolation including the enhanced interactions between the CTCs and the functionalized surface and dynamic flows that prohibit nonspecific binding [51]. Classifications of current CTC enrichment and detection technologies are presented in Figure 2.

## 3. CTCs Detection in HCC Patients and Its Clinical Relevance

By now, many researchers have tried to detect CTCs in patients with HCC and described their relevance to clinical outcome using various approaches, among which EpCAM-based methods are still the most widely employed [46, 52]. The area under curve (AUC) for EpCAM^mRNA+^ CTCs in discrimination between patients with HCC and the others (healthy volunteers, patients with hepatitis B and/or cirrhosis, and patients with benign tumor) was 0.697, with sensitivity of 42.6% and specificity of 96.7%. In particular, when combined with AFP level, the diagnostic value of CTCs was significantly improved and the AUC was 0.857 with sensitivity of 73.0% and specificity of 93.4% [52]. Besides EpCAM, we have initially carried out the aptamer-based “CTC-chip” to capture CTCs in HCC patients with a satisfactory capture ratio (~61%) [51]. With regard to other methods, Vona et al. [44] firstly used the isolation by size of epithelial tumor cell (ISET) method in 2004 to detect CTCs in 44 cases of HCC patients and explored their clinical diagnostic value. The study showed that CTCs and circulating tumor microemboli (CTM) were detected in 52% (23/44) of HCC patients, while, in healthy volunteers, chronic active hepatitis or cirrhosis patients were not detected.

Furthermore, the numbers of CTCs and CTM were also closely correlated with tumor infiltration, portal vein thrombosis, and Child-Pugh class grade B/C level and predicted a poor prognosis of HCC patients [44, 52–57]. EpCAM^mRNA+^ CTC was the only independent prognostic factor for time to recurrence (TTR) of HCC patients [52]. Patients with preoperative detectable EpCAM^mRNA+^ CTCs had significantly shorter TTR (median, 10.9 months versus not reached) and higher recurrence rates (59.6% versus 25.7%) than those without detectable EpCAM^mRNA+^ CTCs. Both the presence and number of CTCs and CTM were significantly associated with a shorter survival [44]. Thus, in order to systematically validate the prognostic value of CTCs detection in HCC patients, we have conducted a meta-analysis of twenty-three published studies [58]. The results show that CTCs' positivity was significantly associated with relapse-free survival (RFS) and overall survival (OS), as well as the characteristics of tumor progression, including TNM stage, vascular invasion, and tumor sites of HCC patients. However, large-scale multicentre trials are still needed to substantiate the claim that CTCs detection will contribute to the future clinical management of HCC patients.

## 4. Characterization of cfNAs

Circulating cell-free nucleic acids (cfNAs) in peripheral blood of cancer patients comprise DNA, mRNA, and miRNAs [59–61]. The earliest report of cfNAs in human peripheral blood was in 1948 [62]; however, cfNAs did not gain attention until 1994, about 50 years later, when studies identified that cfNAs could reflect the RAS gene mutations of cancer patients and thus serve as a kind of tumor markers [63, 64].

## 5. Circulating Cell-Free DNA (cfDNA)

Normal cells can actively secrete nucleic acids to the peripheral blood in the physiological state, cfDNA in the presence of normal human peripheral blood at an average concentration of 30 ng/mL (0–100 ng/mL) [65]. The content of cfDNA in peripheral blood is equivalent to 5000 genomes [66], as a genomic DNA content was approximately 6.6 pg [67, 68]; thus, the peripheral blood is like a “liquid biopsy samples library” which contains large amounts of genetic information. Normal tumor cells and apoptotic and necrotic tumor cells in the tumor microenvironment are considered to be the major source of tumor-related cfDNA in blood of cancer patients [5, 69]. Apoptotic and necrotic tumor cells are usually phagocytized by macrophages or other scavenger cells which can release digested DNA into the circulating system [70]. Compared with healthy subjects, HCC patients contained large amount of tumor-related cfDNAs which are not affected by the size of the tumor and only associated with the degree of malignancy [71], and the amount of circulating DNA is negatively associated with the 3-year DFS and OS of HCC patients [72].

cfDNA is mainly metabolized by the liver, spleen, and kidney [5, 73], and its half-life is just about 4 min to several hours. Therefore, the dynamic level of tumor-related cfDNA reflects the average state of the tumor genome molecular features, which include mutations, copy number variation, methylation abnormalities, microsatellite instability, and loss of heterozygosity, in peripheral blood of cancer patients [74, 75]. Qualitative and quantitative analysis of these changes will help to assess the biological characteristics of the tumor.

## 6. Detection of cfDNA Mutation and Copy Number Variation in Peripheral Blood

Detection of specific gene mutation in peripheral blood is an area of firstly trying to explore the potency of cfDNA as a kind of tumor markers. For now, studies have confirmed that KARAS, EFGR, TP53, and APC are common tumor specific mutations in peripheral blood of patients with solid tumors [14–17, 76–78]. Tumor-suppressive gene TP53 mutation frequently occurs in patients with HCC [14, 15]; 50% of HCC patients living in areas exposed to aflatoxin have tumor suppressor gene P53 (R249S) point mutation, which means amino acid at 249 site AGG changes to AGT [16]. More importantly, point mutation of TP53 can be detected in cfDNA even in the early stage of HCC which is the only gene mutation that can be detected in the patient's circulatory system and is not observed in normal population [17]. Furthermore, patients with AG/GG at rs894151 or AC/CC at rs12438080 in circulating DNA were significantly associated with a decreased OS and TTR of HBV-related HCC patients after liver transplantation [79]. However, with the fact that the probability of gene mutation is relatively low and the enormous content of cfDNA in peripheral blood, thus, the detective method should have high sensitivity and specificity. Furthermore, the complicated extraction process can easily cause cfDNA loss, which will result in increased rate of false-negative results. So, detective methods of cfDNA mutation with ability to overcome these problems will rapidly extend the application of cfDNA in clinic.

DNA copy number variations (CNVs) are distributed very commonly in the human genome and affect more than 10% of the genomic sequence [80]. Furthermore, in tumor patients, CNVs are common genomic changes which have a relatively stable and wide coverage and thus can be used to identify cancer susceptibility loci [18, 19, 81–83]. The commonly amplified regions in HCC are at 1q, 8q, 7q, 17q, and 20q, and the common deleted regions are at 4q, 8p, 13q, 16q, and 17p [20–22], such as CHD1L (1q21) [23] and DLC1 (8p22) [24]. Chan et al. confirmed the existence of typical DNA CNVs in the peripheral blood of 4 hepatocellular carcinoma patients by using massively parallel sequencing, and they almost all disappeared after surgical treatment [19]. Furthermore, oncogenes or antioncogenes usually exist in these regions containing CNVs, which means CNVs detection in these regions has great potency in the early diagnosis of HCC.

## 7. DNA Methylation

DNA methylation is an important epigenetic phenomenon, methylated sites are distributed globally on about 80% of CpGs islands [84], and 5′-end of the promoter region in 60% of the human genes contains CpG islands, the methylation of which plays an important role in regulating gene expression [85]. Changes of DNA methylation widely existed in tumors and played an important role in tumor progression, which is generally characterized by a wide range of genomic hypomethylation and hypermethylation [86–88]. Abnormal methylation occurs before cellular canceration and throughout all stages of malignant transformation, which have the characteristics of tissue specificity and long-term stability; thus, detection of aberrant gene methylation could serve as a potentially powerful approach for early diagnosis of tumors, such as lung cancer and breast cancer [89–91]. The most commonly used detective method is improved and methylation-specific PCR method [92, 93], which is relatively quick and easily performed, and it shows high sensitivity and accuracy to meet the clinical needs for methylation research and application.

Different from genes mutation, many studies have shown that the majority of methylated genes can be detected in the circulatory system of HCC patients, and the cfDNA methylation profile showed good consistency with methylation profile in tumor tissues [25–27, 94, 95]. Iyer et al. [25] detected the methylation statuses of APC, FHIT, p15, p16, and E-cadherin gene in 28 cases of HCC patients and found that methylated rates of these five genes in peripheral blood significantly and positively correlated with the methylated rates in tumor tissues and could well reflect the methylation status of abnormal genes in tumor tissues.

Detection of peripheral DNA methylation has great potential application in the diagnosis, prognosis, and efficacy evaluation of HCC, but the most concerning point is its diagnostic value [28–30, 96]. The Ras association domain family protein 1A (RASSF1A) is a tumor suppressor gene and frequently lost in human cancers by promoter-specific methylation [28, 96]. Chan et al. [28] showed that RASSF1A gene hypermethylation could be detected in the serum of 93% of HCC patients and 58% of the HBV carriers, while only 8% of healthy people had RASSF1A gene hypermethylation. More importantly, correlation analysis further identified that higher concentration of methylated fragments in serum predicted a shorter relapse-free survival time of HCC patients. HCC patients seropositive for the methylated cyclin D2 gene (>70 pg/mL serum) also exhibited a significantly shorter DFS period than patients who were seronegative for the methylated cyclin D2 gene [97].

Given the fact that a single gene methylation showed limited value in the diagnosis of HCC, combined detection of multiple genes methylation status is an effective way to improve the efficiency of diagnosis [31]. The sensitivity and specificity of a combined methylation detection of APC, RASSF1A, GSTP1, and SFRP1 in peripheral blood of HCC patients are 92.7% and 81.9%, respectively [31]. Therefore, in order to improve the sensitivity of early diagnosis of HCC, search for more methylation-related genes involved in HCC progression has great potency in HCC diagnosis.

By now, as summarized in Table 1, multiple alterations in cfDNAs have been found in HCC patients. In the future, with the rapid development of digital PCR and gene sequencing technologies, we believe that the clinical detection of cfDNA variations in cancer patients will become achievable. More importantly, these technologies also provide an effective way for the discovery of additional cfDNA markers.

## 8. Circulating miRNA

Besides cfDNA, there is a certain level of circulating miRNA existing in the peripheral blood. miRNAs are a class of highly conserved noncoding single-stranded endogenous small molecule RNAs, typically 20–25 nt [98]. They are involved in multiple bioactivities of mammalian cells, including stem-cell self-renewal, cellular development, differentiation, proliferation, and apoptosis [99]. In the peripheral blood, endogenous mature miRNA mainly existed in the form of particle by binding with protein or lipoprotein to increase its stability and thus could withstand room temperature, repeated freezing and thawing, acid-base environment, DNA or RNA enzyme treatment, and also other complicated environmental factors [100, 101]. miRNA expression profiling in different cancer types has distinct tissue specificity and expression levels and significantly correlates with tumor classification, diagnosis, and disease progression [102, 103]. Thus, the existence of circulating miRNAs in the peripheral blood of cancer patients has raised the possibility that circulating miRNAs may serve as a kind of novel diagnostic or prognostic markers.

## 9. Detection of Circulating miRNA in HCC Patients and Its Clinical Relevance

As in HCC, although there is no clear miRNA expression profiling, studies have confirmed that several circulating miRNAs closely relate to the progression of HCC, mainly including miR-122, miR-200a, miR-21, miR-223, let-7f, and miR-155 [32–43, 104] (Table 2).

These circulating miRNAs showed great potency for clinical diagnosis of HCC. Yamamoto et al. [39] initially evaluated miRNAs expression in the serum of 10 patients with HCC and demonstrated that miR-500 is an oncofetal miRNA which was abundantly expressed in the sera of HCC patients and returned to normal after the surgical treatment. Zhang et al. [34] further identified that circulating miR-143 and miR-215 both have good diagnostic efficiency in human HCC; the sensitivity and specificity of miR-143 were 78% and 64% and those of miR-215 were 78% and 89%. Given the limited diagnostic value of a single circulating miRNA to HCC, some researchers combined circulating miRNA with AFP to diagnose HCC. The sensitivity and specificity of single miR-21 diagnosis are 60% and 83%, respectively, and the area of 95% confidence interval under ROC curve was 0.69–0.86. However, when combined with AFP, the sensitivity was 81%, specificity of 77%, the area of 95% confidence interval under the ROC curve was 0.74–0.90, significantly higher than the traditional markers AFP (0.54–0.82), and the diagnostic efficiency was greatly improved [33]. The early diagnosis of HCC is clinically desirable, as the prognosis of HCC is significantly improved if the patients get therapy early on. Though circulating miRNAs could be used as prognostic biomarkers for various types of cancers, including colorectal cancer and hematologic cancers [105] and study has confirmed that serum high level of miR-221 expression was correlated with tumor size, cirrhosis, and tumor stage of HCC [38], the prognostic value of circulating miRNAs in HCC is still less reported, which may be owing to the fact that the 5-year overall survival rate of HCC is still only 0%–10%.

Currently, circulating miRNAs' detection is mostly based on quantitative PCR (qPCR); however, before translation into clinical application, it still requires further steps to standardize and validate the technical process employed to measure circulating miRNAs by qPCR. Compared with qPCR, digital PCR (dPCR) has many advantages, which gets the absolute expressive level of miRNA without the standard curve and is not affected by variations caused from samples and PCR amplification efficiency [106]. In lung cancer diagnosis, detection of plasma miRNAs by digital PCR could quantify low abundance of miRNAs with high reproducibility and has showed high sensitivity and specificity in distinguishing lung cancer patients from cancer-free subjects [107, 108]. The application of dPCR in HCC diagnosis is still less reported but it provides a strong guarantee for further development of circulating miRNAs as diagnostic or prognostic biomarkers.

## 10. Conclusion and Outlook

Numerous genetic and epigenetic alterations contribute to oncogenesis and cancer progression, and the progression of cancer is characterized by high heterogeneity. Tumors acquire new mutations that render them resistant to the therapies that target specific genetic mutations. Thus, to improve the clinical outcomes in malignant tumors including HCC, a comprehensive surveillance system comprised early and accurate diagnosis, and dynamic tumor monitoring is necessary. In this regard, a liquid biopsy may capture the entire heterogeneity of tumors. What is more, tumor genotypes are notoriously unstable and prone to changes under selection pressure; thus, liquid biopsies showed great advantages compared to tissue biopsies with regard to the clinical risks of tumor patients and cost, as well as the feasibility of taking serial samples in order to monitor tumor genomic changes in real time.

In terms of liquid biopsy, CTCs have been the most studied as they provide information of tumor progression at both the genetic and cellular levels, while CTCs are relatively rare and require sensitive collection and enrichment technology. cfNAs are emerging as an effective alternative to CTCs, with the benefits of easier collection and analysis. With the expanded application of digital PCR and 3rd-generation sequencing technologies, cfNAs-based detection of liquid biopsies has great potency in early diagnosis and prognostic evaluation of tumors. The personalized information comprised in cfNAs could enable physicians to make more systematic and precise medical decisions.

However, in the past few years, experimental design and detective methods of CTCs and cfNAs in the studies are quite different, with relatively small sample size, which resulted in low stability and comparability in the experimental data across studies. Standardization of detective methods will be a key factor to ensure consistency in clinical application. With the standardization in blood collection and processing and storage and DNA extraction and quantification and analysis and reporting of data, CTCs and cfNAs existing in liquid biopsy might serve as promising detective biomarkers in therapeutic monitoring, prognostic evaluation, and risk assessment of HCC.

## Figures and Tables

**Table 1:** Summary of the role and findings for circulating DNA in HCC.

Type of alteration	Gene	Location	Biomarker	References
Single nucleotide mutations	TP53	17p13.1	Diagnosis	[14–16]
	CTNNB1	3p21	Predictor (exposure to different viral and environmental factors)	[17]

Copy number variation	NA	1p/1q/8p/8q	Diagnosis	[18]
	NA	7q/8q/13q/14p	Diagnosis	[19]
	NA	1q, 8q, 7q, 17q, 20q	Diagnosis	[20–22]
	NA	4q, 8p, 13q, 16q, 17p	Diagnosis	[20–22]
	CHD1L	1q21	Diagnosis	[23]
	DLC1	8p22	Diagnosis	[24]

Methylation changes	APC	5q21-q22	Diagnosis	[25, 26]
	P15/P16	9p21	Diagnosis	[25, 27]
	RASSF1A	3p21.3	Diagnosis	[28]
	TRG5	16q22.1	Diagnosis	[29]
	HOXA9	7p15.2	Diagnosis	[30]
	FHIT	3p14.2	Diagnosis	[25]
	E-caherin	16q22.1	Diagnosis	[25]
	GSTP1	11q13.2	Diagnosis	[31]
	SFRP1	8p11.21	Diagnosis	[31]

NA: not available.

**Table 2:** miRNAs found deregulated in hepatocellular carcinoma.

miRNA	Deregulation	Method	Number of subjects	Reference
miR-1, miR-25, miR-92a, miR-206, miR-375, and let-7f	Up	Solexia sequencing, qRT-PCR	120 HCC, 135 HBV, 48 HCV, and 210 controls	[32]
miR-21	Up	qRT-PCR	126 HCC, 30 CHB, and 50 healthy subjects	[33]
miR-143 and miR-215	Up	qRT-PCR/TaqMan miRNA assays	95 HCC, 118 hepatitis carriers, and 127 controls	[34]
miR-155	Up	qRT-PCR	10 HCV-HCC, 34 HCV, 12 NASH, and 7 healthy subjects	[35]
miR-15b and miR-130b	Up	qRT-PCR	57 HCC, 29 HBC, and 30 controls	[36]
miR-125b	Up	BioMark*™* Dynamic Array	20 HBV-positive HCC, 24 CHB, 22 HBV-positive cirrhosis, and 28 controls	[37]
miR-221, miR-222, and miR-224	Up	qRT-PCR	46 HCC and 20 controls	[38]
miR-500	Up	qRT-PCR	10 HCC	[39]
miR-16 and miR-199a	Down	qRT-PCR	105 HCC patients, 107 CLD, and 71 controls	[40]
miR-122	Down	qRT-PCR	62 HCC, 48 CLD, and 34 controls	[41]
miR-29	Down	qRT-PCR	17 HCC and 17 controls	[42]
miR-152	Down	qRT-PCR	89 HCC and 89 controls	[43]
miR-200a	Down	qRT-PCR	41 HCC and 41 controls	[43]
miR223-3p	Down	BioMark Dynamic Array	20 HBV-positive HCC, 24 CHB, 22 HBV-positive cirrhosis, and 28 controls	[37]

CHB: chronic hepatitis B; NASH: nonalcoholic steatohepatitis; HBC: hepatitis B carriers; CLD: chronic liver diseases.
